# *Fusobacterium nucleatum* downregulated MLH1 expression in colorectal cancer by activating autophagy-lysosome pathway

**DOI:** 10.3389/fimmu.2025.1586146

**Published:** 2025-05-19

**Authors:** Tingting Ding, Minkang Wu, Li Zhao, Hu Liu, Xuanke Cao, Jing Guo, Xingchen Zhu, Lamei Zhao, Heping Zhang, Yaohui Gao, Qing Wei

**Affiliations:** ^1^ Department of Pathology, Shanghai Tenth People’s Hospital, Tongji University School of Medicine, Tongji University, Shanghai, China; ^2^ Department of Medical Oncology, Jinling Hospital, Affiliated Hospital of Medicine School, Nanjing University, Nanjing, China; ^3^ Department of General Surgery, Shanghai Tenth People’s Hospital, Tongji University School of Medicine, Tongji University, Shanghai, China; ^4^ Department of Radiology, Dongfang Hospital, Beijing University of Chinese Medicine, Beijing, China; ^5^ Department of Hematology, Shanghai East Hospital, Tongji University School of Medicine, Shanghai, China

**Keywords:** colorectal cancer, *Fusobacterium nucleatum*, MLH1 deficiency, autophagy-lysosome pathway, immunotherapy

## Abstract

**Backgrounds:**

*Fusobacterium nucleatum* (*F. nucleatum*) has been shown to be associated with immunotherapy in colorectal cancer (CRC), but its exact mechanism needs to be further explored.

**Methods:**

We first analyzed the correlation between *F. nucleatum* abundance and mismatch repair (MMR) protein deficiency in CRC tissues from 567 patients. We then treated CRC cells and tissues with *F. nucleatum* and its metabolites. RNA sequencing was used to evaluate the involved pathways, and non-targeted metabolomics was employed to analyze the metabolites regulating MLH1. CRC cells were treated with butyrate, a metabolite of *F. nucleatum*, with or without the autophagy-lysosome pathway inhibitor chloroquine or mTOR activator MHY1485. Finally, subcutaneous tumors of BALB/C mice were treated with PD-L1 blockade, butyrate, or their combination.

**Results:**

The results showed that the abundance of *F. nucleatum* in CRC tissues is correlated with MSI and MLH1 deficiency. *F. nucleatum*, its culture supernatant, and its metabolite butyrate cause the downregulation of MLH1 protein via autophagy-lysosome pathway. Subcutaneous tumors in mice received the combined treatment of PD-L1 blockade and butyrate shrink more evidently than those disposed by single therapy.

**Conclusions:**

*F. nucleatum* reduces MLH1 expression via the lysosomal pathway by butyrate, leading to deficient mismatch repair (dMMR), which may yield therapeutic benefits in CRC patients with microsatellite stability (MSS).

## Introduction

According to statistics on 36 cancers in 185 countries worldwide in 2020, colorectal cancer (CRC) is among the most prevalent life-threatening cancers. The prognosis for metastatic CRC is even more dismal, with a 5-year survival rate of 14% (1). Therefore, there is an urgent need to develop alternative and effective treatments for patients with this disease.

The application of immune checkpoint inhibitors, especially PD-1/PD-L1 inhibitors, has shed light on the outcomes of CRC. Both the CheckMate-142 and KEYNOTE-164 trials have shown that advanced CRC patients with microsatellite instability (MSI) or deficient mismatch repair (dMMR) have a much higher response rate to PD-1 inhibitors than those with microsatellite stability (MSS) or proficient mismatch repair (pMMR) (2, 3). However, patients with MSI/dMMR only account for approximately 15% of the entire CRC population and less than 5% of metastatic CRC (4). Further surveys are necessary to improve the effectiveness of this novel strategy in a wider range of patients.

Although it is believed that MSI/dMMR occurs in the initiation process of CRC, there are also studies suggesting that exogenous stimuli might alter MSI/dMMR status during cancer development. For instance, Mackay et al. reported a loss of MLH1 protein in patients after chemotherapy (5), which raised the intriguing question of whether MSS/pMMR CRC could be converted into MSI/dMMR CRC, thus overcoming primary resistance to immunotherapy.

*Fusobacterium nucleatum* (*F. nucleatum* or Fn), a gram-negative anaerobic bacterium that mainly inhabits the oral or gastrointestinal tract, participates in various biological processes of CRC development through direct contact with its secreted metabolites. In addition, clinical studies have shown that *F. nucleatum* is associated with MSI/dMMR in CRC. Tahara et al. found that Fusobacterium in CRC tissue is related to molecular characteristics such as CIMP, *TP53* wild-type, *MLH1* promoter methylation, MSI, and *CHD*7/8 mutations (6). Chia et al. reported that dMMR CRC has a higher abundance of *F. nucleatum* than pMMR CRC (7), which is consistent with the findings of Yu et al. showing more enriched *F. nucleatum* in MSI CRC tissue than in MSS ones (8). Nevertheless, the above data were all from clinical observations, and whether there is a direct or indirect regulatory relationship between *F. nucleatum* and MSI/dMMR remains to be determined.

In the current study, we aimed to investigate whether and how *F. nucleatum* affects the dMMR and modulates anti-PD-L1 treatment responses in CRC. We found that the abundance of *F. nucleatum* in CRC tissues correlated with MSI and MLH1 deficiency. Exploring its mechanism, we found that *F. nucleatum* can metabolize butyric acid, which further reduces the expression of MLH1 through the lysosomal pathway, leading to dMMR. Our study further supports the mechanism by which *F. nucleatum* can enhance the efficacy of immunotherapy.

## Materials and methods

### Tissue samples

Formalin-fixed paraffin-embedded (FFPE) tissue blocks were collected from 567 patients who had undergone surgery or colonoscopy at the Shanghai Tenth People’s Hospital and were pathologically diagnosed with CRC between December 2018 and April 2020. Medical records were reviewed, and the clinicopathological and molecular characteristics of these CRC patients, including age, sex, smoking history, family history, tumor differentiation, tumor size, tumor location, and Ki-67 expression, and molecular characteristics, including MSI/*KRAS*/*BRAF V600E* status, mismatch repair (MMR) proteins, p53, and EGFR, were retrospectively acquired. TNM staging was determined according to the 8th edition of the American Joint Committee on Cancer (AJCC). The study was approved by the Institutional Review Board of Shanghai Tenth People’s Hospital.

### Quantification of *F. nucleatum* in CRC tissues

Genomic DNA was extracted from FFPE tissue blocks of 567 primary CRCs using the QIAamp DNA FFPE Tissue Kit according to the manufacturer’s instructions (Qiagen, Valencia, CA, USA). Quantitative real-time PCR was performed in 10 μl reactions including TB Green Premix Ex Taq (Takara, Japan), primers, and 500 ng template DNA on a 7500 PCR Instrument under the following reaction conditions: 10 min at 95°C, followed by 40 cycles of denaturation at 95°C for 15 s and at 60°C for 1 min. The primers used for *F. nucleatum* were as follows:

*F. nucleatum* forward primer:
5’-CAACCATTACTTTAACTCTACCATGTTCA-3’;
*F. nucleatum* reverse primer:
5’-GTTGACTTTACAGAAGGAGATTATGTAAAAATC-3’.
*F. nucleatum* abundance was defined as the CT value relative to the reference gene *PGT*.

### Cell culture

CRC cell lines, including COLO 205, SW480, DLD1, and CT26, were purchased from the American Type Culture Collection (ATCC; Manassas, VA, USA). COLO 205, DLD1, and CT26 were cultured in RPMI1640 (Gibco, USA), whereas SW480 cells were cultured in DMEM (Gibco, USA) supplemented with 10% fetal bovine serum (FBS) (Gibco, USA) and penicillin/streptomycin in a humidified incubator with 5% CO2 at 37°C.

### Bacterial strain, culture conditions and *ex-vivo* infection experiment

*F. nucleatum* subsp. nucleatum ATCC 25586 and *F. nucleatum* subsp. nucleatum isolated from fresh CRC tissues were cultured in fluid Thioglycollate Medium (agar-free) in an anaerobic environment at 37°C. The supernatant of *F. nucleatum* was collected and filtered with a 0.4 μm filter after centrifugation at 4000 rpm for 10 min. For infection experiment, *F. nucleatum* in exponential phase [OD600 (optical density at 600 nm) ranged from 0.4~0.6] was added to CRC cells cultured without penicillin/streptomycin at a multiplicity of infection (MOI) of 1000 and the supernatant of *F. nucleatum* was added to the cell medium at a ratio of 1:10 for 12 hours.

### RNA extraction and quantitative real-time PCR

Total RNA was extracted from CRC cells using TRIzol reagent, following the manufacturer’s instructions (Invitrogen, Carlsbad, CA, USA). cDNA was synthesized with 1 μg of total RNA in a 10 μl system using the Takara PrimeScript One Step RT-PCR Kit (Takara, Japan). Quantitative real-time PCR (qRT-PCR) was performed on an Applied Biosystems 7500 quantitative PCR system (Applied Biosystems, Foster City, CA) for 10 min at 95°C, followed by 40 cycles of 5 s at 95°C, 30 s at 95°C, and 30 s at 60°C in per cycle. Each sample was tested in triplicate. The 2^-ΔΔCT^ method was used to determine the relative expression of the target genes. *Actin* and *GAPDH* were used as reference genes for human and mouse CRC cells, respectively.

### RNA sequencing

RNA extraction, database construction, sequencing, and bioinformatics analyses were performed by Shenzhen Huada Gene Co. Ltd.

### Western blotting

Tissue and cellular proteins were extracted using RIPA Lysis Buffer (Strong) (Beyotime Biotech. Inc. Shanghai, China) and quantified using the BCA Protein Assay Kit (Thermo Scientific, USA). Proteins were separated by 8% sodium dodecyl sulfate polyacrylamide gel electrophoresis (SDS-PAGE) and then transferred onto 0.4 µm nitrocellulose membranes (GE Healthcare). The membrane was blocked with 5% fat-free milk in TBST for 1 h at room temperature, followed by incubation with the corresponding primary antibodies overnight at 4°C and a secondary antibody at room temperature for 2 h. Probe proteins were detected using the Immobilon Western Chemilum HRP substrate (Millipore, Germany).

### DNA methyltransferase activity detection

DNA methyltransferase (DNMT) activity was detected using the DNMT1/3A/3B Assay Kit (Abcam, UK) according to the manufacturer’s instructions. Briefly, nuclear extraction, dilution buffer, capture antibody, detection antibody, and DNMT standard dilution were prepared and successively added to the corresponding plates. The OD value was measured at a wavelength of 450 nm. The DNMT activity was estimated according to the formula provided.

### Metabolite detection

The metabolites within the *F. nucleatum* culture supernatant and control medium were detected by non-targeted metabolomics testing by Shanghai Ouyi Biomedical Technology Co., Ltd. Liquid chromatography-mass spectrometry (LC-MS) includes sample pretreatment, metabolite extraction, and LC-MS full scan detection. The gas chromatography-mass spectrometry (GC-MS) detection process includes sample pretreatment, metabolite extraction, metabolite derivatization, and GC-MS detection.

### Stimulating CRC cells with the metabolites of *F. nucleatum*

Short chain fatty acids (SCFAs) were used to stimulate CRC cells with a concentration of 1, 2, 5, 10, and 100 mM for 3 to 24 hours. Chloroquine (CQ) and MHY1485 were added to the cell medium 2 h before SCFAs at 10 μM.

### Animal experiments

Thirteen 4-week-old nude mice, housed under standard conditions, were divided into two groups. COLO 205 cells (5 × 10^6^ COLO 205 cells were subcutaneously injected into the right flank of mice. Tumor volume was monitored every three days. Once they reached 100m^3^, 1 × 10^9^ *F. nucleatum* in 100 μl phosphate buffer saline (PBS) and 100 μl PBS were injected into the subcutaneous tumor tissue every two days, respectively. After 2 weeks, the tumors were harvested.

Four-week-old BALB/c mice were purchased from Shanghai Lingchang Biotechnology Co., Ltd. (Shanghai, China) at 4 weeks old. After one-week of acclimation, the mice were implanted with 5 × 10^5^ CT26 cells. and randomized into four groups for treatment with PBS and anti-PD-L1 isotype IgG (control), PBS and PD-L1 inhibitor (100 μg per mouse), sodium butyrate (50 mg/kg dissolved in 100 μl PBS), anti-PD-L1 isotype IgG, or a combination of PD-L1 inhibitor and sodium butyrate (SB). One week after implantation, mice were treated intraperitoneally with vehicle, PD-L1 inhibitor, SB, or a combination of PD-L1 inhibitor and SB. Mouse subcutaneous tumor tissues were frozen in liquid nitrogen or fixed in formaldehyde at the end of the experiment. All mouse experiments were approved by the Ethics Committee of the Shanghai Tenth People’s Hospital (SHSY-IEC-4.1/19–180/02).

### Statistical analysis

Continuous variables between two and three different groups were examined using Student’s t-test and One-way ANOVA, respectively. Cases with detectable *F. nucleatum* were divided into high-or low/negative groups based on the median cut-off amount of *F. nucleatum* DNA. The correlation between *F. nucleatum* DNA load and categorical clinicopathological variables was assessed using the chi-squared test. Univariate and multivariate logistic regression models were used to analyze the statistical interaction between MMR protein expression and the clinicopathological characteristics of CRC. A backward stepwise elimination with a threshold of p< 0.1 in the univariate logistic regression model was used to select variables for the multivariate models. Statistical significance was defined as a two-tailed p-value<0.05 was considered statistically significant. All statistical analyses were performed using SPSS23.0 and GraphPad Prism 8.0.

## Results

### The relationship between *F. nucleatum* abundance and clinicopathological and molecular characteristics of CRC tissues

We first reviewed the medical records of patients admitted to the Department of Gastrointestinal Surgery at Shanghai Tenth People’s Hospital from December 2018 to March 2021 and collected the clinicopathological and molecular information of patients who were pathologically diagnosed with colorectal carcinoma (**Supplementary Table S1**).

**Figure 1:**
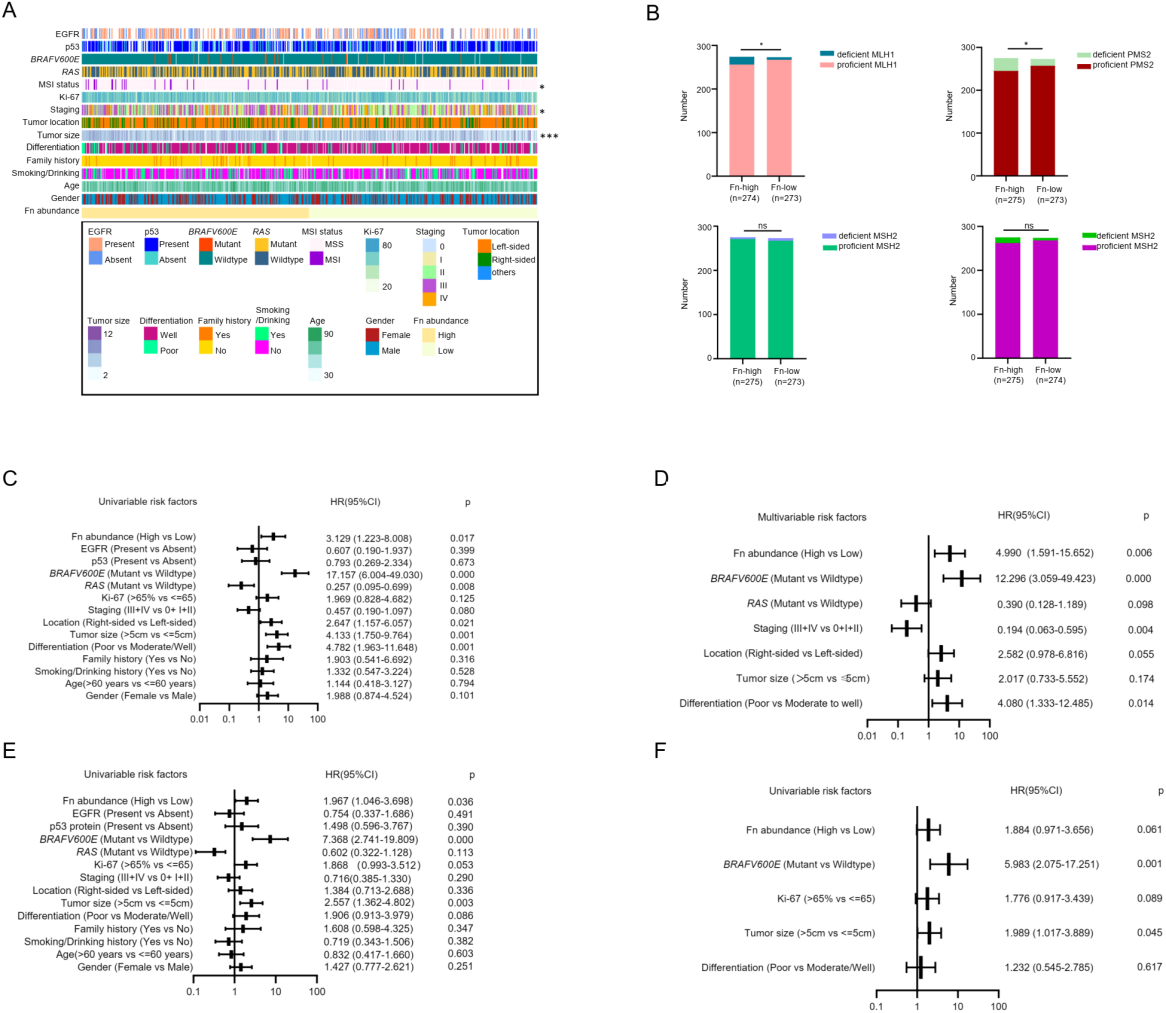
High *F. nucleatum* abundance in tumor tissue is associated with MSI and MMR protein deficiency. **(A)** The correlation between *F. nucleatum* abundance in tumor tissue and clinicopathological and molecular features of CRC patients displayed by heatmap. High DNA level of *F. nucleatum* is related to MSI status, tumor staging, and tumor size. Statistics method: chi-square test. **(B)** The correlation between *F. nucleatum* abundance and mismatch repair (MMR) protein expression. High abundance of *F*. *nucleatum* is related to deficient MLH1 and PMS2, but not to MSH2 and MSH6. Statistics method: chi-square test. **(C)** Univariate analysis of risk factors for MLH1 deficiency. High *F nucleatum* abundance, right-sided tumor, tumor size more than 5 cm, poor differentiation and BRAFV600E mutation are risk factors for absent MLH1 expression, while RAS mutation is a protective factor for MLH1 deficiency. **(D)** Multivariate analysis of risk factors for MLH1 deficiency. High *F*. *nucleatum* abundance, poor differentiation, BRAFV600E mutation are risk factors for the loss of MLH1 expression. Advanced clinical stage (III+IV) is a protective factor for MLH1 expression deletion. **(E)** Univariate analysis of risk factors for PMS2 deficiency. Enriched *F. nucleatum* in the CRC tissue, BRAFV600E mutation, and tumor size more than 5 cm are risk factors for PMS2 deficiency. **(F)** Multivariate analysis of risk factors for PMS2 deficiency. BRAFV600E mutation and tumor size more than 5 cm are risk factors for PMS2 expression loss. *p <0.05, ns: not significant.

We then dichotomized the cases into *F. nucleatum* -low and *F. nucleatum* -high groups based on the median cut point CT value of *F. nucleatum* DNA relative to the reference gene *PGT* assessed by the quantitative PCR assay. We then used the chi-squared test to analyze the correlation between *F. nucleatum* DNA levels and the clinicopathological and molecular features of patients. The results showed that *F. nucleatum* abundance was associated with tumor size (p<0.001), tumor staging (p<0.05), and MSI status (p<0.05) (**Figure 1A**). We further explored the association between *F. nucleatum* richness and T, N, and M stages. *F. nucleatum* abundance was only related to N staging, in addition to T and M staging (**Supplementary Figure S1A**).

As it has been confirmed that four MMR proteins (MLH1, MSH2, MSH6, and PMS2) determine MSI status in humans (9), we assumed that *F. nucleatum* might be related to MMR protein expression. To confirm this hypothesis, we evaluated MMR protein levels in CRC patients evaluated by immunohistochemistry (IHC). MMR protein expression was scored as absent or present by two experienced pathologists according to CAP guidelines, which declared that any confirmed positive staining in the tumor cell nucleus was identified as proficient MMR expression unless the tumor cell nucleus had completely negative staining. Representative negative and positive samples for MLH1, MSH2, MSH6, and PMS2 protein expression are shown in **Supplementary Figure S1B**. In our study, 52 of 535 (9.7%) CRC patients with dMMR were observed, among whom the most frequent deficiency was the concomitant loss of MLH1 and PMS2 (details are shown in **Supplementary Figure S1C**). Analysis of the relationship between *F. nucleatum* and MMR protein expression indicated that *F. nucleatum* abundance was associated with the absence of MLH1 and PMS2 expression, instead of MSH2 and MSH6 proteins (**Figure 1B**).

To identify the risk factors for MLH1 and PMS2 deficiency, we performed univariable and multivariable ordinal logistic regression analyses using four MMR proteins as dependent variables and various clinicopathological and molecular features as well as *F. nucleatum* abundance as independent variables. The logistic regression model included gender (female vs. male), age (>60 years vs. ≤ 60 years), smoking/drinking history (Yes vs. No), family history (Yes vs. No), differentiation (Poor vs. Moderate/Well), tumor size (>5 cm vs. ≤ 5 cm), tumor location (Right-sided vs Left-sided), tumor staging (III + IV vs. 0 + I + II), Ki-67 (>65% vs. ≤ 65%), *RAS* status (Mutant vs. Wildtype), *BRAFV600E* status (Mutant vs. Wildtype), p53 protein (Present vs. Absent), EGFR protein (Present vs. Absent), and *F. nucleatum* abundance (High vs. Low). Univariable logistic regression analysis for MLH1 deficiency showed that high *F. nucleatum* abundance (HR=3.129, 95% CI: 1.223-8.008, p=0.017), *BRAFV600E* mutation (HR=17.157, 95% CI: 6.004-49.030, p<0.001), right-sided (HR=2.647, 95% CI: 1.157-6.057, p=0.021), tumor size > 5 cm (HR=4.133, 95% CI: 1.750-9.764, p=0.002), and poor differentiation (HR=4.782, 95% CI: 1.963-11.648, p=0.001) were risk factors, whereas *RAS* mutation was a protective factor (HR=0.257, 95% CI: 0.095-0.699, p=0.008) for the absent expression of MLH1 (**Figure 1C**). To evaluate the independent risk factors, we further included the independent variables with p-value less than 0.1 from the univariate logistic regression analysis into the multivariate logistic regression equation to remove confounding factors. The results revealed that the high abundance of *F. nucleatum* (HR=4.990, 95% CI: 1.963-11.648, p=0.001), *BRAFV600E* mutation (HR=12.296, 95% CI: 3.059-49.423, p<0.001), and poor differentiation (HR=4.080, 95% CI: 1.333-12.485, p=0.014) were independent risk factors, and advanced stage (III + IV) was a protective factor (HR=0.194, 95% CI: 0.063-0.595, p=0.004) for MLH1 deficiency (**Figure 1D**).

Next, the same calculation is applied to PMS2. Univariate logistic regression analysis revealed that high *F. nucleatum* abundance (HR=1.967, 95% CI: 1.046-3.698, p=0.036), *BRAFV600E* mutation (HR=7.368, 95% CI: 2.741-19.809, p<0.001), and tumor size > 5 cm (HR=2.557, 95% CI: 1.362-4.802, p=0.003) were risk factors for PMS2 deficiency (**Figure 1E**). In multivariate logistic regression analysis, *BRAFV600E* (HR=5.983, 95% CI: 2.075-17.251, p=0.001) and tumor size > 5 cm (HR=1.989, 95% CI: 1.017-3.889, p=0.045) were independent risk factors for absent PMS2 expression. *F. nucleatum* abundance was excluded (HR=1.884, 95% CI: 0.971-3.656, p=0.061) (**Figure 1F**). In contrast, the abundance of *F. nucleatum* did not correlate with MSH2 or MSH6 expression in univariable or multivariable logistic regression analyses (**Supplementary Figures S1D, E**).

### *F. nucleatum* induced downregulation of MLH1 protein in *in-vivo* and *in-vitro* experiments

The above analysis indicated that *F. nucleatum* was associated with MSI and was an independent risk factor for MLH1 protein deficiency; however, it was unclear whether *F. nucleatum* induced MSI/dMMR. Therefore, we conducted *in-vivo* and *in-vitro* experiments to clarify this question.

**Figure 2:**
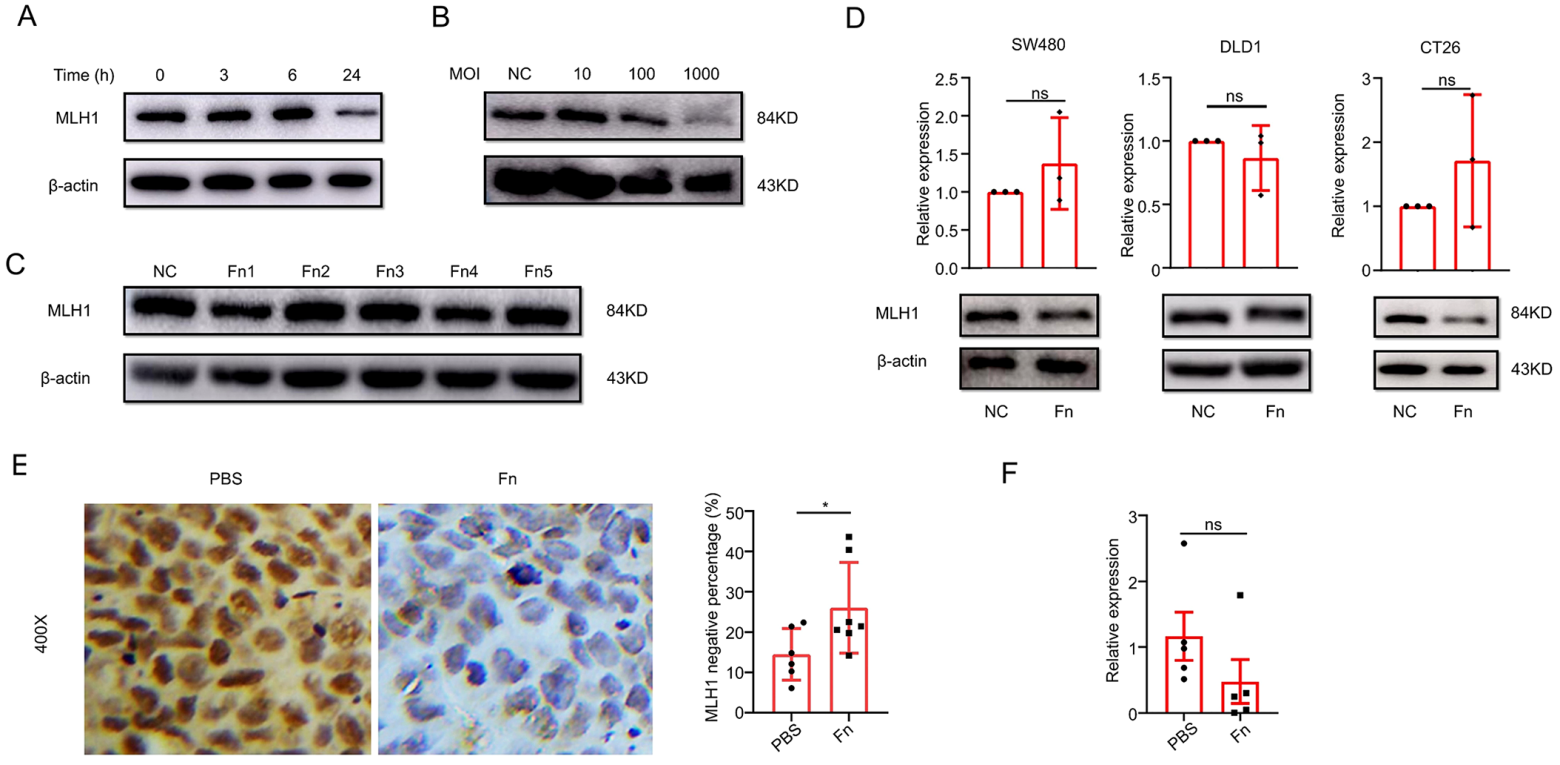
*F. nucleatum* infection induces MLH1 protein downregulation in ex-vivo and *in-vivo* experiments. **(A)** MLH1 protein levels at different times after *F. nucleatum* infection in CRC cell line COLO 205. **(B)** MLH1 protein levels in CRC cell line COLO 205 at the multiplicity of infection (MOI) of 10, 100, and 1000, respectively after *F. nucleatum* infection for 24 hours. **(C)** The effect of different *F. nucleatum* subspecies isolated from CRC tissue on the expression levels of MLH1 protein in COLO 205. **(D)** MLH1 protein is downregulated compared to the control group in wild-type CRC cell lines SW480, DLD1, and CT26 after being infected by *F. nucleatum* for 24 hours at the MOI of 1000, but the mRNA levels of MLH1 in the three CRC cell lines do not change compared to the control group. **(E)** In the mouse subcutaneous tumor model, immunohistochemistry shows that the percentage of MLH1 negative cells is significantly higher in tumor tissues administrated with *F. nucleatum* than that injected with PBS. **(F)** The mRNA level of MLH1 in subcutaneous tumor samples of *F. nucleatum* group is not significantly different from that of the PBS group. NC, negative control. *p <0.05, ns: not significant.

We co-cultured the MSS CRC cell line COLO 205 with *F. nucleatum* (ATCC 25586) for different periods (0, 3, 6, and 24 h) and different multiplicities of infection (MOI) (10, 100, 1000). We found that *F. nucleatum* reduced MLH1 protein expression most remarkably after co-culturing for 24 h (**Figure 2A**) and at an MOI of 1000 (**Figure 2B**). Moreover, various *F. nucleatum* subspecies isolated from the cancer tissues of CRC patients had a role in downregulating MLH1 expression of COLO 205 at an MOI of 1000 for 24 h (**Figure 2C**). This study also analyzed the influence of *F. nucleatum* on MLH1 expression in wild-type human MSS CRC cell line SW480, MSI CRC cell line DLD1, and mouse MSS CRC cell line CT26. The results showed that MLH1 protein was decreased in all three cell lines after stimulation with *F. nucleatum* under the same conditions as indicated by western blotting, while the mRNA levels of these CRC cell lines were not significantly different between the *F. nucleatum* -infected group and the control group (**Figure 2D**).

Next, we constructed a nude mouse subcutaneous tumor model to explore the effect of *F. nucleatum* on MLH1 protein expression. COLO 205 (1×10^7^) was subcutaneously injected into the right armpit of the nude mice. When the volume of the subcutaneous tumor reached approximately 100mm^3^, 10^9^ CFU of *F. nucleatum* was dissolved in 100 μl PBS and an equivalent volume of PBS was injected into the tumors every two days, respectively. Two weeks later, the subcutaneous tumors were removed, and IHC was used to detect MLH1 protein expression in the tumor tissue. The staining results showed that compared with the PBS group (n=6), the MLH1 protein expression in the tumor tissue of the *F. nucleatum* group (n=7) decreased significantly (p<0.05) (**Figure 2E**), but the *MLH1* mRNA level remained unchanged (**Figure 2F**). These *in-vivo* and *in-vitro* results demonstrate that *F. nucleatum* decreases MLH1 protein expression in CRC tissues at the post-transcriptional level.

### *F. nucleatum* culture supernatant downregulated MLH1 expression

**Figure 3:**
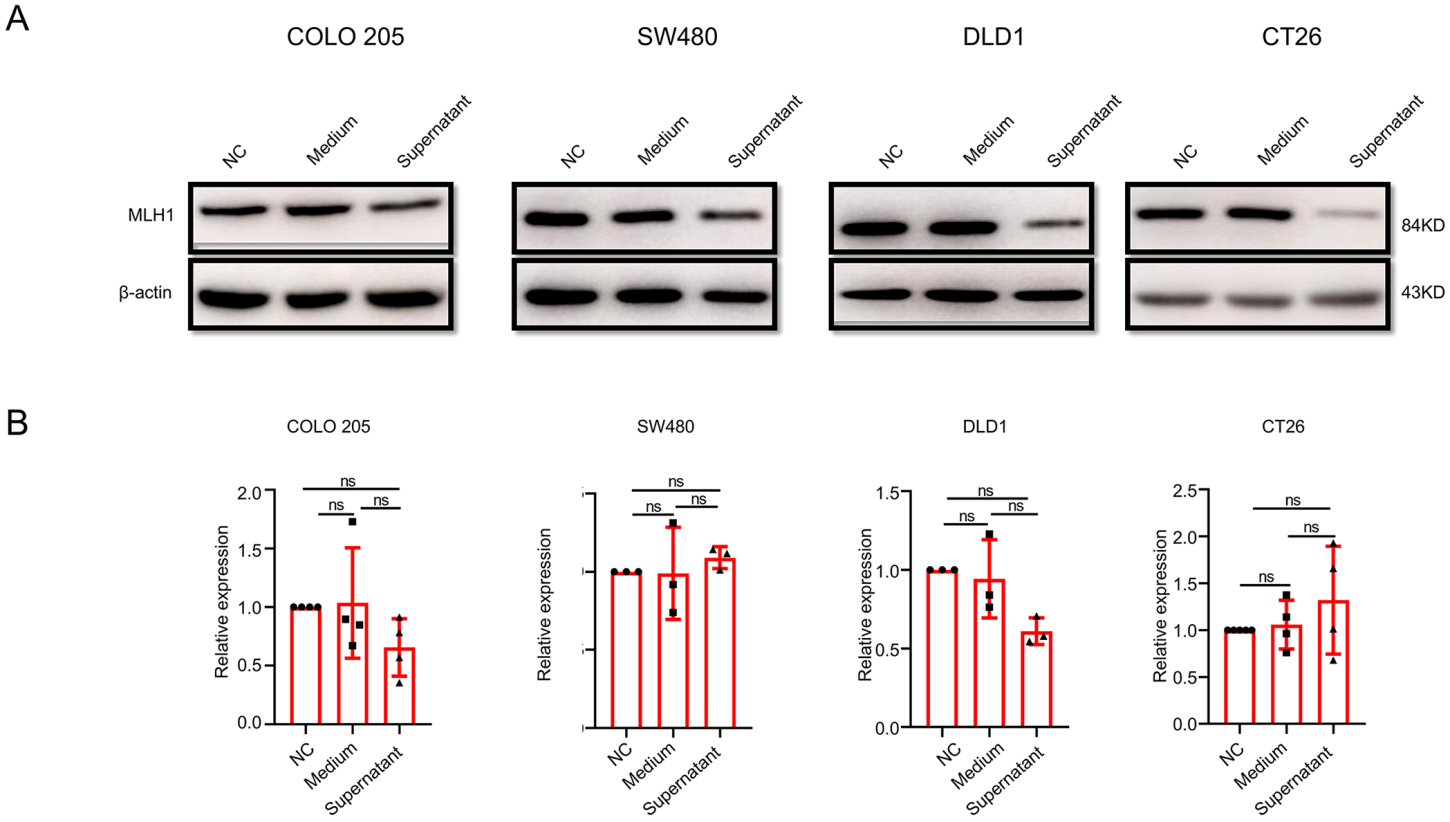
The influence of *F. nucleatum* culture supernatant on MLH1 protein and mRNA expression in different wild-type CRC cell lines. **(A)** Compared with that from the control group and blank medium infected group, cells stimulated by *F. nucleatum* culture supernatant expresses reduced MLH1 protein. **(B)** The *F. nucleatum* culture supernatant has no effect on MLH1 mRNA compared with the control group and the blank medium group. ns: not significant; NC: negative control. Medium: blank medium; Supernatant: Fn culture supernatant.

*F. nucleatum* performs various functions through a series of metabolites. To clarify whether the downregulation of MLH1 by *F. nucleatum* is mediated by its metabolites, we collected blank medium and culture supernatant to stimulate CRC cells for 12 h and detected MLH1 protein expression. The results showed that compared to the control group, MLH1 expression in COLO 205, SW480, DLD1, and CT26 cells in the blank medium cells did not change but was significantly downregulated in the *F. nucleatum* culture supernatant cells (**Figure 3A**), indicating that the regulation of MLH1 protein by *F. nucleatum* is at least partially mediated through the metabolites in the culture supernatant. Next, we analyzed the changes in *MLH1* mRNA levels in CRC cell lines stimulated with *F. nucleatum* culture supernatant. Our data showed that there was no statistically significant difference in *MLH1* mRNA expression between the control, blank medium, and *F. nucleatum* supernatant groups (**Figure 3B**). This suggests that the regulation of the MLH1 protein in CRC cells also occurs at the post-transcriptional level.

### Downregulation of MLH1 expression by the short chain fatty acids

**Figure 4:**
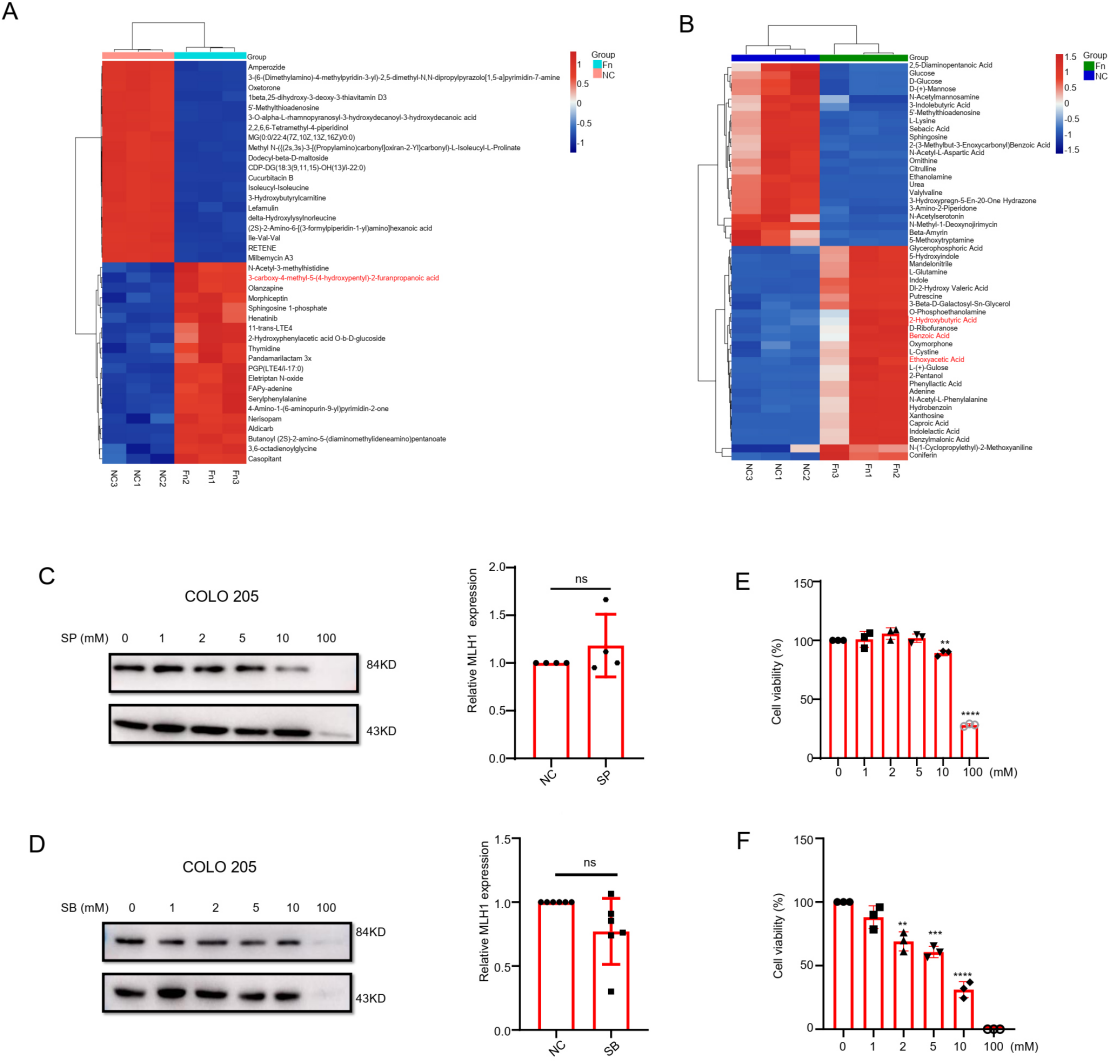
The role of short chain fatty acids (SCFAs) on the expression of MLH1. **(A)** Liquid chromatography-mass spectrometry (LC-MS) result indicates that furanopropionic acid is elevated in *F. nucleatum* culture supernatant compared with the blank medium group. **(B)** The gas chromatography-mass spectrometry (GC-MS) test shows that benzoic acid, ethoxyacetic acid, and 2-hydroxybutyric acid are upregulated in *F. nucleatum* culture supernatant group compared with the blank medium group. **(C, D)** COLO 205 cells are stimulated with different concentrations (0–100 mM) of Sodium Propionate (SP) and Sodium Butyrate (SB) for 24 hours. The results show that SP and SB downregulate MLH1 protein expression from 10–100 mM and 1–100 mM, respectively. When stimulated COLO 205 cell with SP (10 mM) and SB (1mM), there is no significant change of the MLH1 mRNA levels compared with their control groups. **(E, F)** The effect of SP and SB on the viability of COLO 205 cells at different concentrations. SP decreased cell viability at 10 and 100 mM, and SB decreased cell viability at 2, 5, 10 and 100 mM. NC: negative control. **p<0.01, ***p<0.001 and ****p<0.0001.

According to previous reports, the metabolites of *F. nucleatum* mainly include short-chain fatty acids (SCFAs), ammonium salts, and H2S, among which SCFAs play an important role in CRC therapy (10–12). Therefore, we further analyzed the differential metabolites in the *F. nucleatum* culture supernatant. The results showed that compared with the blank medium, the upregulated differential metabolites in *F. nucleatum* culture supernatant were mainly organic acids and their derivatives, lipids and lipid-like molecules, organic heterocyclic compounds, nucleosides/nucleotides, and their analogs. Propionic acid was one of the main differential metabolites detected by LC-MS (**Figure 4A**), and formic acid, acetic acid, and butyric acid were among the differential metabolites detected by GC-MS (**Figure 4B**). Considering the important role that SCFAs play in tumor development, we stimulated the human CRC cell line COLO 205 with various concentrations of SCFAs. We found that at concentrations ranging from 0 to100 mM, sodium formate (SF) had no remarkable effect on the expression of MLH1 in cells, whereas sodium acetate (SA) decreased MLH1 protein at a high concentration (100 mM) (**Supplementary Figure S2A**). In contrast, sodium propionate (SP) and SB downregulated MLH1 expression at 10–100 and1–100 mM respectively, at the protein level instead of the mRNA level (**Figures 4C, D**). Notably, SB (1 mM) did not significantly inhibit the proliferation of COLO 205 cells (**Figure 4E**), but SP (10 mM) influenced cell viability (**Figure 4F**). These results suggest that low concentrations of SB may regulate the expression of MLH1 in CRC cells under physiological rather than pathological conditions.

Except for COLO 205, we also observed downregulation of MLH1 protein in SW480, DLD1, and CT26 cells stimulated by SB at a concentration of 1 mM (**Supplementary Figure S2B**). However, MLH1 mRNA levels did not change between cells treated with SB (1 mM) and those in the control group (**Supplementary Figure S2C**).

### *F. nucleatum* induced MLH1 protein degradation via autophagy-lysosome pathway

In sporadic MSI-CRC, MLH1 deficiency is often caused by promoter methylation. Although our data suggested that the regulation of MLH1 protein by *F. nucleatum* occurs at the post-transcriptional level, in order to rule out the possible co-existing mechanism, we still tested *MLH1* promoter methylation degree, which turned out to be 6%, 10%, 8%, 10%, and 10% in the whole blood DNA of normal individuals, the control COLO 205 cells (NC), the blank medium treated cells (Medium), *F. nucleatum* supernatant stimulated cells (supernatant), and *F. nucleatum* infected cells (Fn), respectively (**Supplementary Figure S3A**). These data suggest that compared to normal cells, the methylation level of *MLH1* promoter in cancer cells was elevated, but treatment with *F. nucleatum* or its culture supernatant did not further exacerbate methylation. In addition, we tested the enzymatic activity of DNMTs. The results showed that *F. nucleatum* and its culture supernatant did not increase the activity of DNMT1 and DNMT3B in COLO 205 cells or the activity of DNMT1, DNMT3A, and DNMT3B in subcutaneous tumor tissue of nude mice infected with *F. nucleatum* (**Supplementary Figures S3B, C**). Moreover, the enzymatic activity of DNMT3B was partially downregulated (**Supplementary Figure S3C**). Our research suggests that the downregulation of MLH1 protein by *F. nucleatum* and its metabolites does not occur via the common *MLH1* promoter methylation pathway.

**Figure 5:**
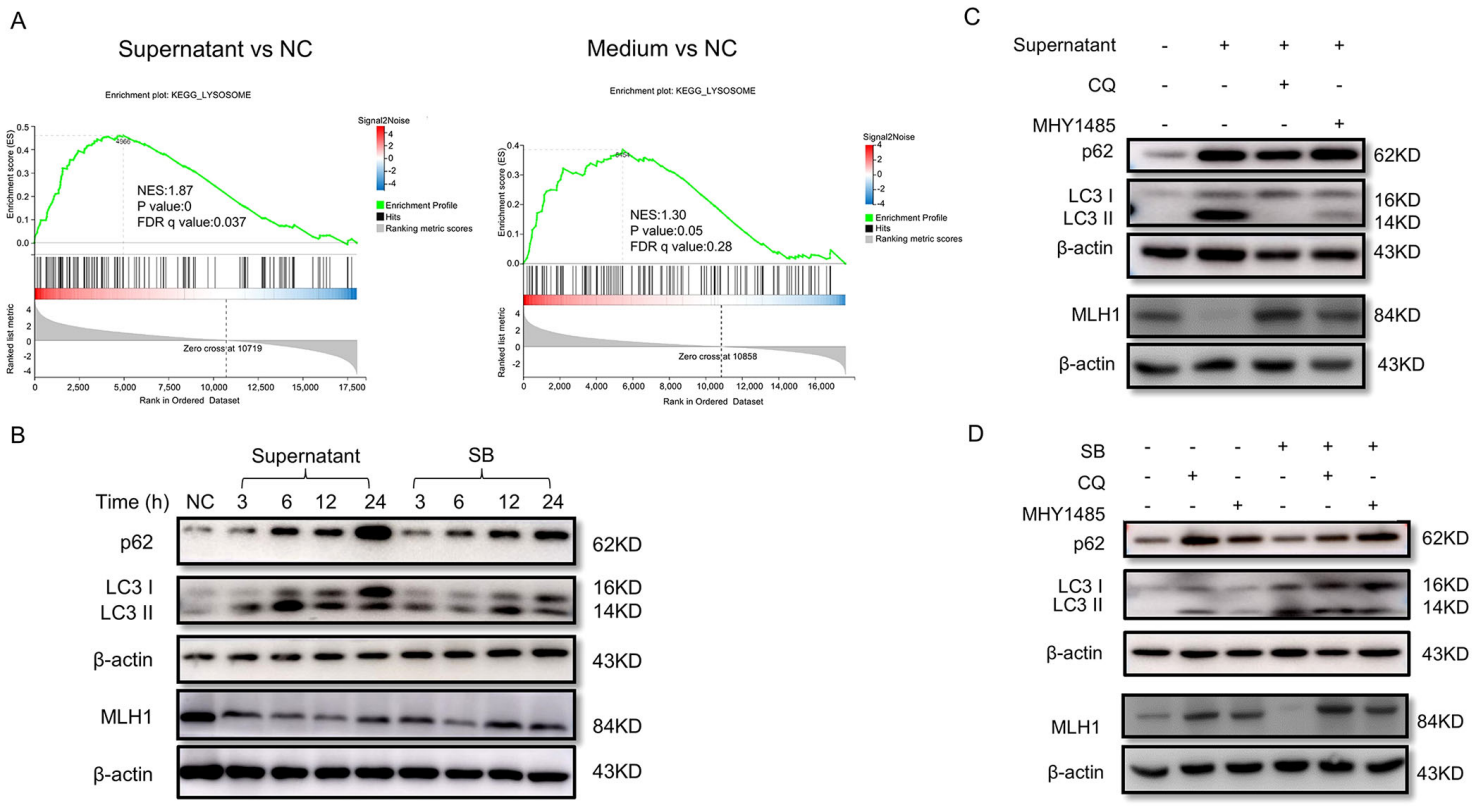
*F. nucleatum* culture supernatant and SB downregulated MLH1 protein via autophagy-lysosome pathway. **(A)** GSEA analysis shows that the lysosome pathway is activated after stimulation with *F. nucleatum* supernatant other than blank medium in COLO 205 cells. **(B)** The variations of autophagy-lysosome markers, LC3II/I and p62, and MLH1 protein in COLO 205 stimulated by *F. nucleatum* culture supernatant and SB at different time points. The expression of MLH1 is downregulated with both treatments. Meanwhile, the ratio of LC3II/I and p62 are increased. **(C)** Western blot shows that COLO 205 cells expressed decreased MLH1 protein with the treatment of the *F. nucleatum* supernatant. After the addition of autophagy inhibitor CQ (10 μM) and the mTOR signaling pathway agonist MHY1485 (10 μM), the downregulation of MLH1 expression induced by *F. nucleatum* supernatant is partly reversed. **(D)** Western blot shows that the addition of SB into COLO 205 cell culture medium at the concentration of 1 mM reduces MLH1 protein level. But when co-treatment with CQ (10 μM) or MHY1485 (10 μM), MLH1 is reversed compared with SB treated group.

We then conducted RNA sequencing with RNA collected from COLO 205 cells of the control, blank medium, and *F. nucleatum* culture supernatant groups to identify the key regulatory molecules or signaling pathways responsible for MLH1 downregulation. Gene Set Enrichment Analysis (GSEA) revealed that the lysosome pathway, an important signaling pathway for protein degradation, was significantly upregulated in the supernatant group compared to the blank medium group (**Figure 5A**). Therefore, we suspect that the culture supernatant of *F. nucleatum* may activate this pathway, leading to MLH1 protein degradation.

To verify this possibility, we first detected the expression levels of LC3II/I and p62, the key proteins of the autophagy-lysosome pathway, in COLO 205 cells under different treatments. The results showed that the ratio of LC3 II/I and p62 were both increased, while MLH1 protein was decreased after *F. nucleatum* supernatant stimulation for 3 to 24 h (**Figure 5B**), indicating that the autophagy-lysosome pathway was activated. Next, we added the autophagy inhibitor CQ (10 μM) or its upstream pathway mTOR agonist MHY1485 (10 μM) into the system two hours before the supernatant to inhibit the autophagy-lysosome pathway. We found that the MLH1 protein levels in the supernatant combined with CQ group, or supernatant with MHY1485 group were partly reversed compared to the groups treated with the supernatant, CQ, or MHY1485 alone (**Figure 5C**). Therefore, we considered that *F. nucleatum* supernatant degraded MLH1 protein by activating the autophagy-lysosome signaling pathway, and that the mTOR signaling pathway may be involved in the upstream pathway.

To determine whether SB regulated MLH1 expression through the autophagy-lysosome pathway, we measured MLH1, LC3 II/I, and p62 expression after SB addition. The results suggested that SB reduced MLH1 protein and increased LC3 II/I and p62 protein levels after stimulation for 3 to 24 h (**Figure 5B**). When co-treated with CQ or MHY1485, the downregulation of MLH1 induced by SB was also partially reversed (**Figure 5D**), indicating that SB also decreased MLH1 via autophagy-lysosomes.

### Sodium butyrate enhanced the efficiency of PD-L1 inhibitor in mouse subcutaneous tumor model

**Figure 6:**
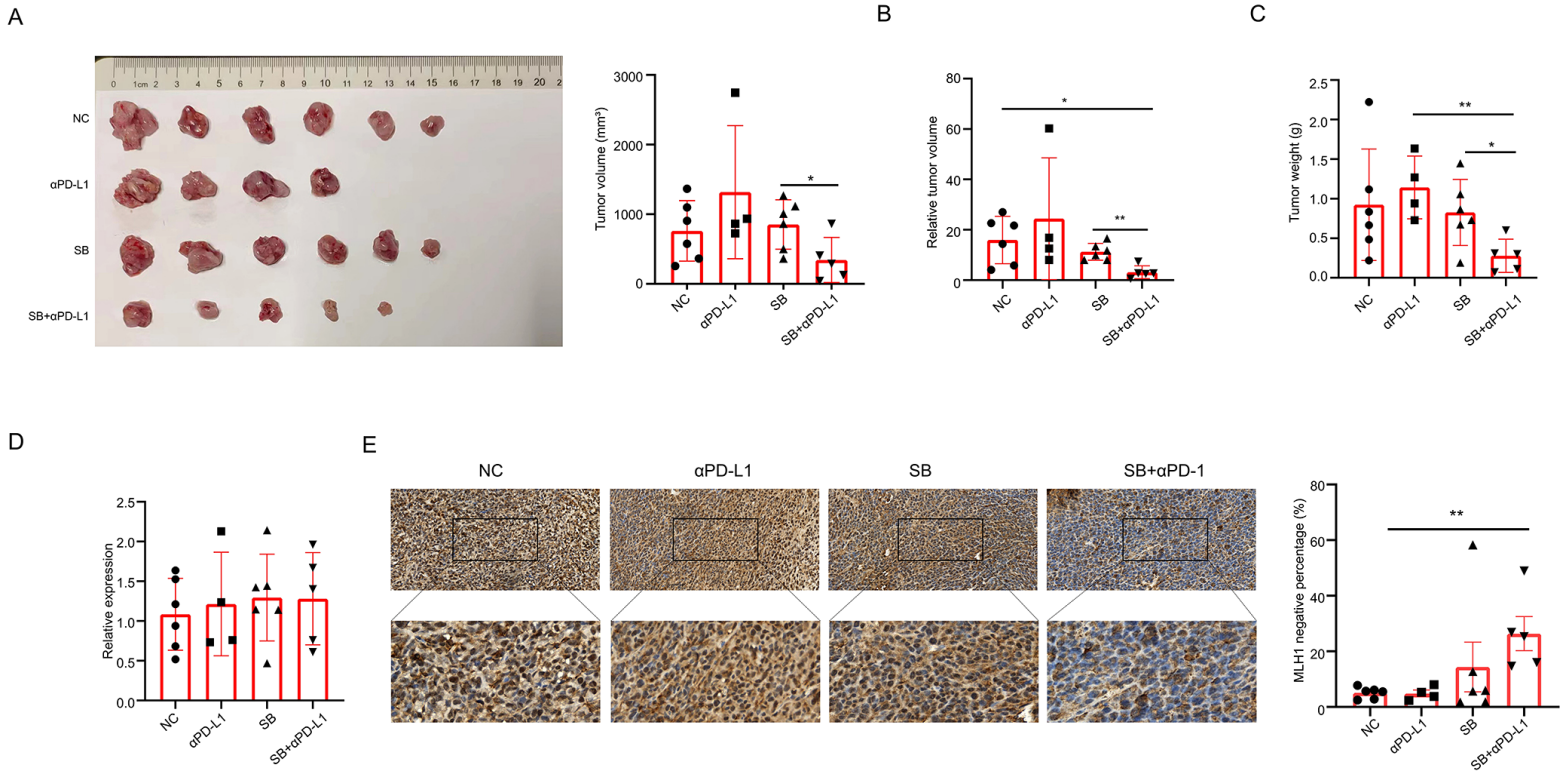
Sodium butyrate enhances the efficacy of PD-L1 inhibitor in subcutaneous tumor mice. **(A, B)** Tumor volume and relative tumor volume in different tumor groups. Tumor volume in SB+αPD-L1 mice is significantly less than that in SB group. Moreover, the relative tumor volume in SB+αPD-L1 mice is less than that in both the control and SB group. **(C)** Tumor weights in different groups. The tumor weight in SB+αPD-L1 group is less than that in the αPD-L1 group and SB group. **(D)** The mRNA levels of MLH1 in different groups are not different. **(E)** Immunohistochemistry indicates that the percentage of MLH1 negative tumor cells is significantly higher in tumor tissues administrated with SB and αPD-L1 than that of the control group. *p<0.05, **p<0.01.

Our *ex-vivo* cell experiments showed that SB downregulated MLH1 protein expression and induced dMMR. As dMMR CRC has a higher response rate to PD-1/PD-L1 blockade than pMMR CRC, we suspected that SB administration improved the efficiency of anti-PD-L1 mAb. Therefore, we constructed a BALB/c tumor-bearing mouse model to test the influence of SB on immunotherapy. At the end of the experiment, the tumor volume, relative tumor volume, and tumor weight of the different groups were assessed. Compared with the SB-treated group, the tumor volume and relative tumor volume of the SB+αPD-L1 group were significantly decreased (**Figures 6A, B**). When comparing the tumor weights, significant differences between the SB+αPD-L1 and αPD-L1 groups or the SB group were observed (**Figure 6C**).

We further detected MLH1 protein and mRNA expression in tumors treated with different treatments. This indicated that mRNA levels did not vary greatly among the four groups (**Figure 6D**), but MLH1 protein expression was significantly reduced in the SB+αPD-L1 group compared with the control group. Although not statistically significant, the ratio of MLH1 negative tumor cells was higher than that of the control and αPD-L1 groups (**Figure 6E**).

**Figure 7:**
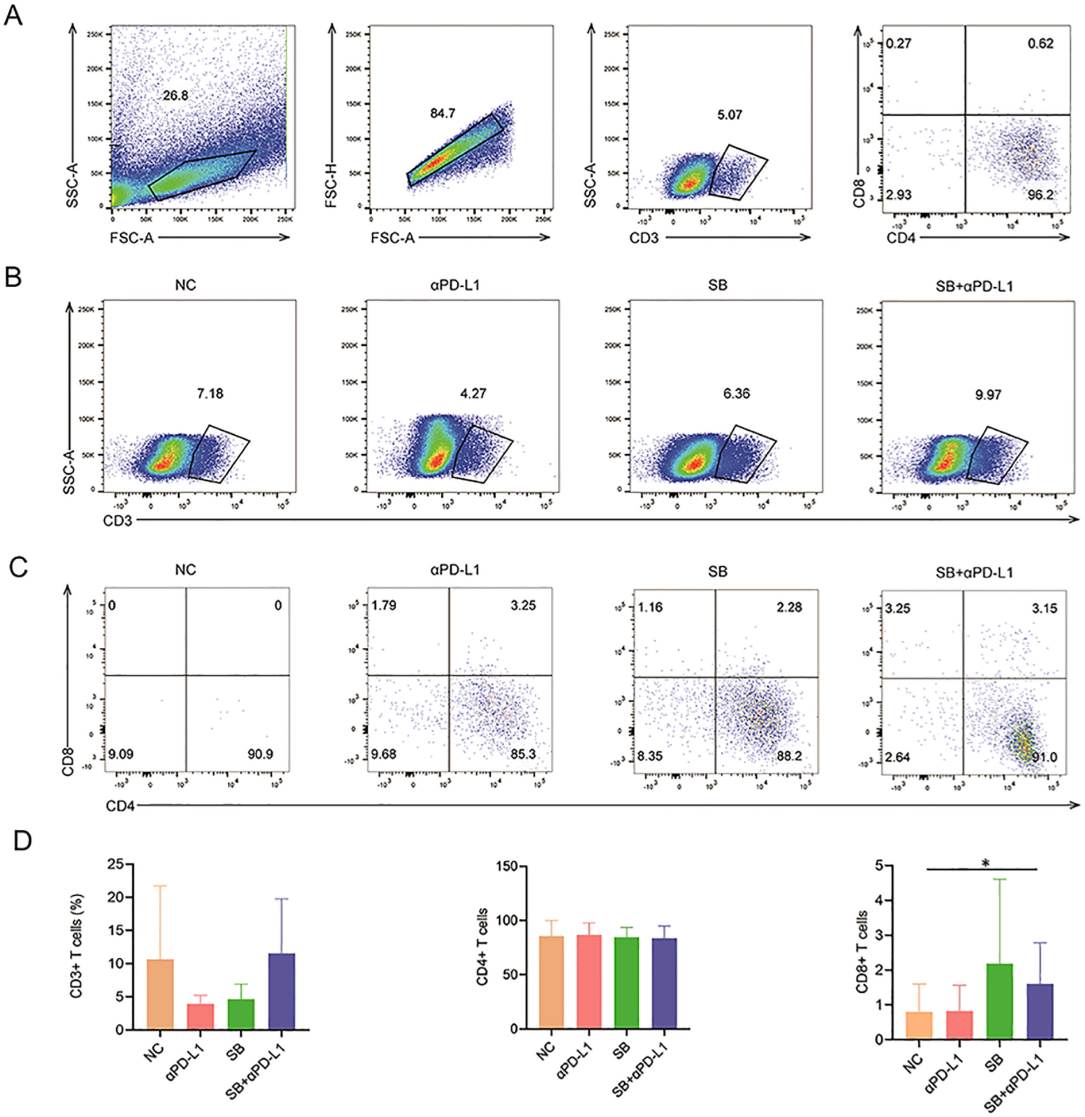
Lymphocyte infiltration in mice with different treatments. **(A)** The gating process of lymphocyte subpopulation. **(B, C)** The ratios of CD3+, CD4+ and CD8+ T cells showed by representative sample of different groups. **(D)** CD3+ and CD4+ T cells in different groups are not significantly different. In contrast, the infiltration CD8+ T cells in tumor tissue of SB+αPD-L1 is remarkably richer than that in the control group. *p<0.05.

It has been documented that dMMR tumors have more lymphocyte infiltration, which favors immunotherapy; therefore, we tested the richness of lymphocytes and various T cell subsets. Compared to the control and αPD-L1 groups, the SB and SB+αPD-L1 groups tended to have higher percentages of CD8+ T cells (**Figure 7D**), despite not having more lymphocytes, CD3+ T cells, and CD4+ T cells (**Figures 7A–C**), suggesting that SB plus αPD-L1 might boost the tumor immune microenvironment.

## Discussion

PD-1/PD-L1 blockade shows promising results in metastatic CRC (mCRC) with MSI but has unsatisfactory efficiency in the majority of the MSS population (13). An attempt to convert MSS/pMMR into MSI/dMMR is required for the broader clinical utility of immunotherapy. This study found that *F. nucleatum*, a gram-negative anaerobic bacterium residing in the oral and gastrointestinal tracts, downregulates the expression of the MMR protein MLH1 in CRC via its metabolite butyrate, resulting in dMMR and sensitizing immunotherapy.

Tahara et al. reported that CRC patients with high levels of Fusobacterium in intestinal cancer tissue are associated with molecular characteristics such as CIMP positivity, *MLH1* promoter methylation, and MSI (6). Okita et al.’s analysis suggests that high abundance of *F. nucleatum* infection in CRC tissue is related to elevated levels of microsatellite alterations in MSI or specific tetranucleotide repeat sequences (14). Consistently, in the present study, we also found a correlation between *F. nucleatum* and MSI. Moreover, further analysis showed that *F. nucleatum* is an independent risk factor for MLH1 deficiency, raising the question of whether this bacterium plays a regulatory role in MLH1. By detecting the levels of MLH1 in CRC cell lines and CRC tissues infected by *F. nucleatum*, we confirmed that it induces downregulation of the MLH1 protein and causes dMMR, heralding the potential to augment the effect of immunotherapy in patients with MSS.

Methylation of *MLH1* promoter is the principal mechanism underlying MLH1 deficiency in sporadic MSI CRC (9). Previous data showed that *F. nucleatum* is not only related to MSI status but also to *MLH1* promoter methylation and DNMT activity (8). Nevertheless, we did not detect variations in these two indicators in CRC cell lines or CRC tissue treated with *F. nucleatum* or its metabolites, excluding the involvement of *MLH1* promoter methylation in MLH1 protein loss. The post-translational modification pathway participates in the regulation of MLH1 expression. Chen et al. found that MLH1 protein can be hydrolyzed by Caspase-3 when cells are treated with tumor necrosis factor-related apoptosis-inducing ligands or the topoisomerase II inhibitor etoposide (15). Daniels’ research suggests that tyrosine kinase inhibitors imatinib and nilotinib cause a decrease in MLH1 protein levels but do not change *MLH1* mRNA levels. Further research has shown that MLH1 is degraded through the lysosomal pathway (16). To test whether *F. nucleatum* regulates MLH1 expression via post-translational modification pathways, we performed RNA sequencing using the COLO 205 cell line stimulated with the supernatant of *F. nucleatum.* Enrichment analysis revealed that the lysosomal pathway is activated. Moreover, the downregulation of MLH1 induced by *F. nucleatum* supernatant was partially restored after the addition of pathway inhibitors, further indicating that the autophagy-lysosomal pathway leads to a decrease in MLH1 protein levels induced by *F. nucleatum* or its metabolite butyrate. For the first time, we proposed that *F. nucleatum* downregulates MLH1 expression and induces dMMR through the autophagy-lysosome pathway.

As previously discussed, combinations of different immunotherapy targets or immunotherapy with chemotherapy/radiotherapy/targeted therapy improve the response rate of MSS CRC to PD-L1 inhibitors (17–21). However, the drawbacks of this method, including increasing adverse effects and cost, cannot be overlooked. Therefore, the development of combined treatments featuring effectiveness, economy, and tolerable safety profile is an active area of investigation.

The conversion of MSS/pMMR to MSI/dMMR by inactivating the MMR gene *MLH1* has been reported to cause cancer regression in mice with normal immune function by altering the immune microenvironment (22). Given that *F. nucleatum* can induce MLH1 loss, it is expected to serve as a potential target for sensitization immunotherapy. However, overall microbiota transplantation has potential risks considering the cancer-promoting effect of *F. nucleatum;* thus, utilizing the components of *F. nucleatum* that are responsible for dMMR seems to be a solution to this dilemma. Previous research has suggested that SCFAs, which are among the *F. nucleatum* metabolites, have a regulatory effect on the immune system (23) and are correlated with immunotherapy (24, 25). Therefore, we analyzed the relationship between SCFAs and the MSI/dMMR. The results of *ex-vivo* and *in-vivo* experiments showed that butyrate, an SCFAs, can induce downregulation of MLH1 protein at low concentrations. Moreover, *in-vivo* experimental data suggested that butyrate combined with PD-L1 inhibitors can enhance the efficacy of immunotherapy. The data further enriches the understanding of the mechanism by which microbes regulate the efficacy of immunotherapy and provide evidence for priming pMMR/MSS CRC sensitive to immunotherapy.

ICIs with butyrate cotreatment have shown some therapeutic potential, but there are still many limitations that should be considered carefully. First, neither *ex-vivo* nor *in-vivo* subcutaneous tumor models can simulate the actual tumor microenvironment; thus, the applicability of butyrate with ICIs needs to be tested in a more rigorous investigation. Second, the toxic effects of butyrate should also be evaluated in depth. Despite these findings, we have proven that *F. nucleatum* can downregulate MLH1 expression through butyric acid, enriching the mechanism of MSI formation, providing a possibility for MSS-to-MSI conversion, and partly explaining the mechanism by which the gut microbiota enhances immunotherapy efficacy. Hopefully, this surge of knowledge will lead to improved pharmacological strategies to overcome primary resistance to immunotherapy in pMMR CRC.

## Data Availability

The RNA-seq datasets presented in this study have been deposited in the NCBI Sequence Read Archive (SRA) under the BioProject accession number PRJNA1259493. These data is accessible starting via the following link: https://www.ncbi.nlm.nih.gov/sra/PRJNA1259493.

## Supplementary material

Supplementary Figure 1 The association of *F. nucleatum* abundance with TNM staging and MSH2 and MSH6 expression. **(A)** The correlation between *F. nucleatum* abundance and TNM staging. High abundance of *F. nucleatum* is related to N staging other than T staging and M staging. **(B)** Immunohistochemical images of positive and negative expression of four MMR proteins in CRC tissue. **(C)** The distribution of four MMR proteins in CRC patients. **(D, E)** Univariate analysis shows that *F. nucleatum* is not a risk factor for MSH2 and MSH6 deficiency.

Supplementary Figure 2 The influences of Sodium Butyrate on MLH1 protein and mRNA in different CRC cell lines. **(A)** COLO 205 cells are stimulated with different concentrations (0–100 mM) of Sodium Formate (SF) and Sodium Acetate (SA) for 24 hours. SA decreases MLH1 expression at the concentration of 100mM, while SF has no influence on MLH1 protein expression from 1–100 mM. **(B, C)** In SW480, DLD1 and CT26 cell line, SB reduces MLH1 protein level at the concentration of 1–100 mM, respectively, but has no effect on MLH1 mRNA level at the concentration 1 mM.

Supplementary Figure 3 Ex-vivo and *in-vivo* experiments are conducted to investigate the effects of *F. nucleatum* and its culture supernatant on MLH1 promoter methylation and DNMT activity in COLO 205 cells. **(A)** The promoter methylation degrees of MLH1 in normal human peripheral blood, untreated COLO 205 cells, blank medium treated COLO 205 cell, supernatant treated COLO 205 cell and *F. nucleatum* infected COLO 205 cells are ≤ 6%, 10%, 8%, 10% and 10%, respectively. **(B)** In ex-vivo experiments, there is no difference in the activity of DNMT1 and DNMT3B between the blank medium and *F. nucleatum* culture supernatant, as well as between *F. nucleatum* stimulated COLO 205 and the control group. **(C)** No variations of the enzymatic activities of DNMT1, DNMT3A, and DNMT3B are found in subcutaneous tumors of nude mice from the PBS group and the *F. nucleatum* infected group. All experiments were independently repeated 3 times. *p<0.05.
