# Supplementary figures and images for: Fusobacterium nucleatum downregulated MLH1 expression in colorectal cancer by activating autophagy-lysosome pathway
Source: Front Immunol. 2025 May 19;16:1586146. doi: 10.3389/fimmu.2025.1586146 (PMC12127293; doi:10.3389/fimmu.2025.1586146)

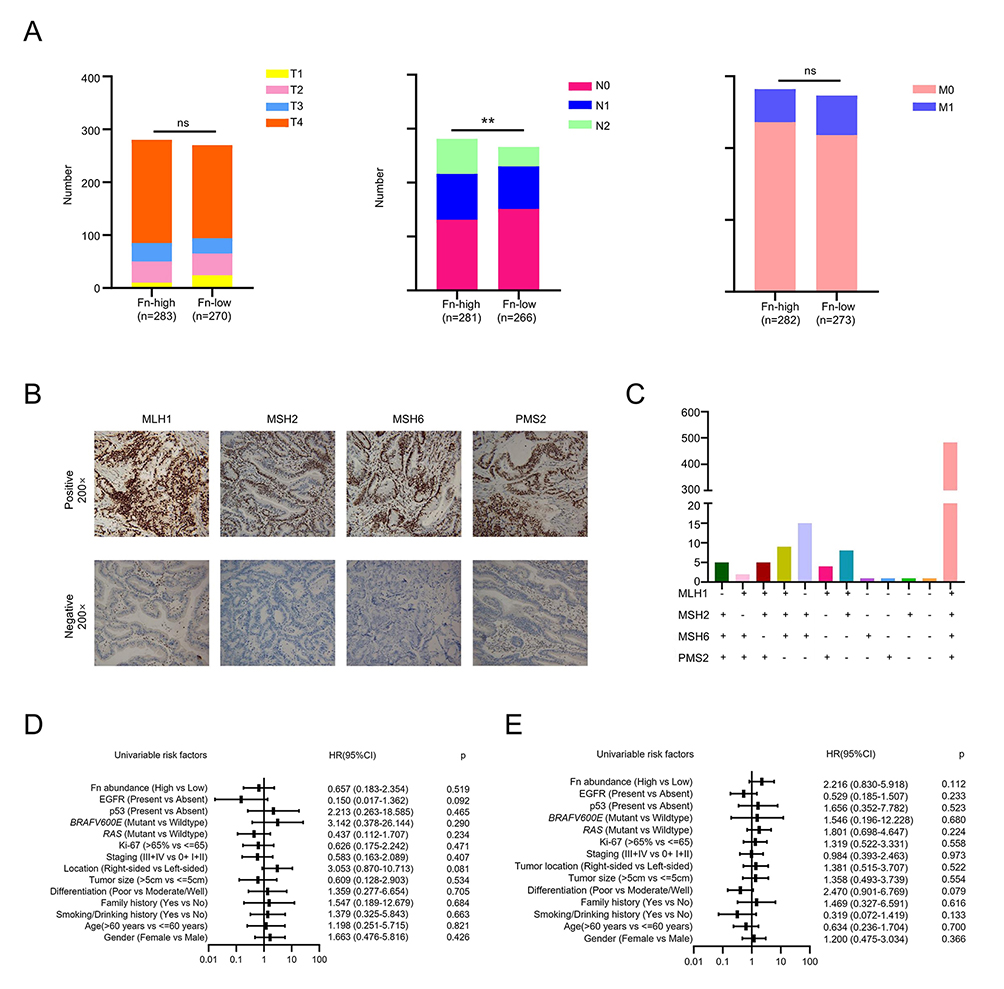

Supplement: Supplementary Figure 1 — The association of F. nucleatum abundance with TNM staging and MSH2 and MSH6 expression. (A) The correlation between F. nucleatum abundance and TNM staging. High abundance of F. nucleatum is related to N staging other than T staging and M staging. (B) Immunohistochemical images of positive and negative expression of four MMR proteins in CRC tissue. (C) The distribution of four MMR proteins in CRC patients. (D, E) Univariate analysis shows that F. nucleatum is not a risk factor for MSH2 and MSH6 deficiency. [file Image1.jpeg]

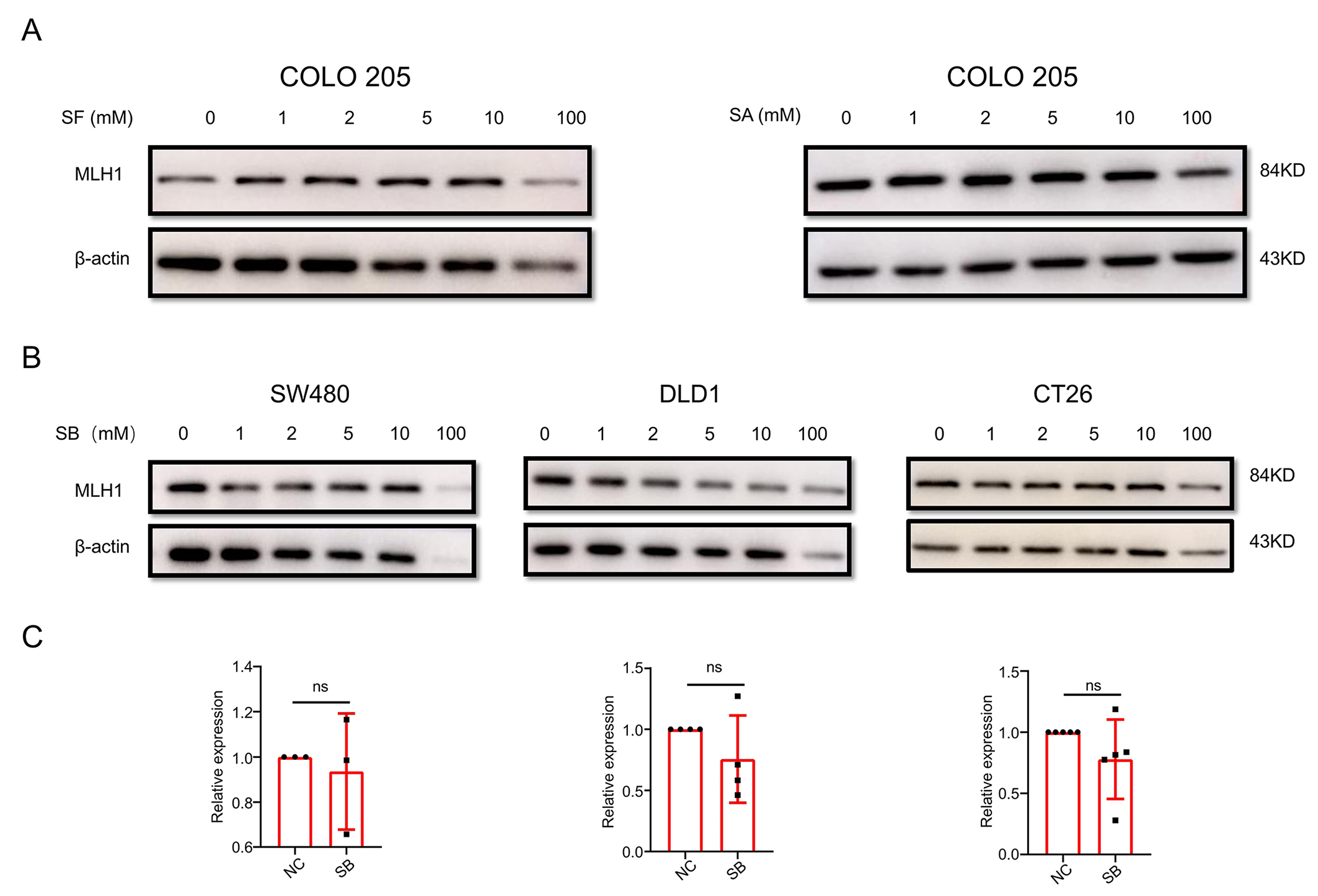

Supplement: Supplementary Figure 2 — The influences of Sodium Butyrate on MLH1 protein and mRNA in different CRC cell lines. (A) COLO 205 cells are stimulated with different concentrations (0–100 mM) of Sodium Formate (SF) and Sodium Acetate (SA) for 24 hours. SA decreases MLH1 expression at the concentration of 100mM, while SF has no influence on MLH1 protein expression from 1–100 mM. (B, C) In SW480, DLD1 and CT26 cell line, SB reduces MLH1 protein level at the concentration of 1–100 mM, respectively, but has no effect on MLH1 mRNA level at the concentration 1 mM. [file Image2.jpeg]

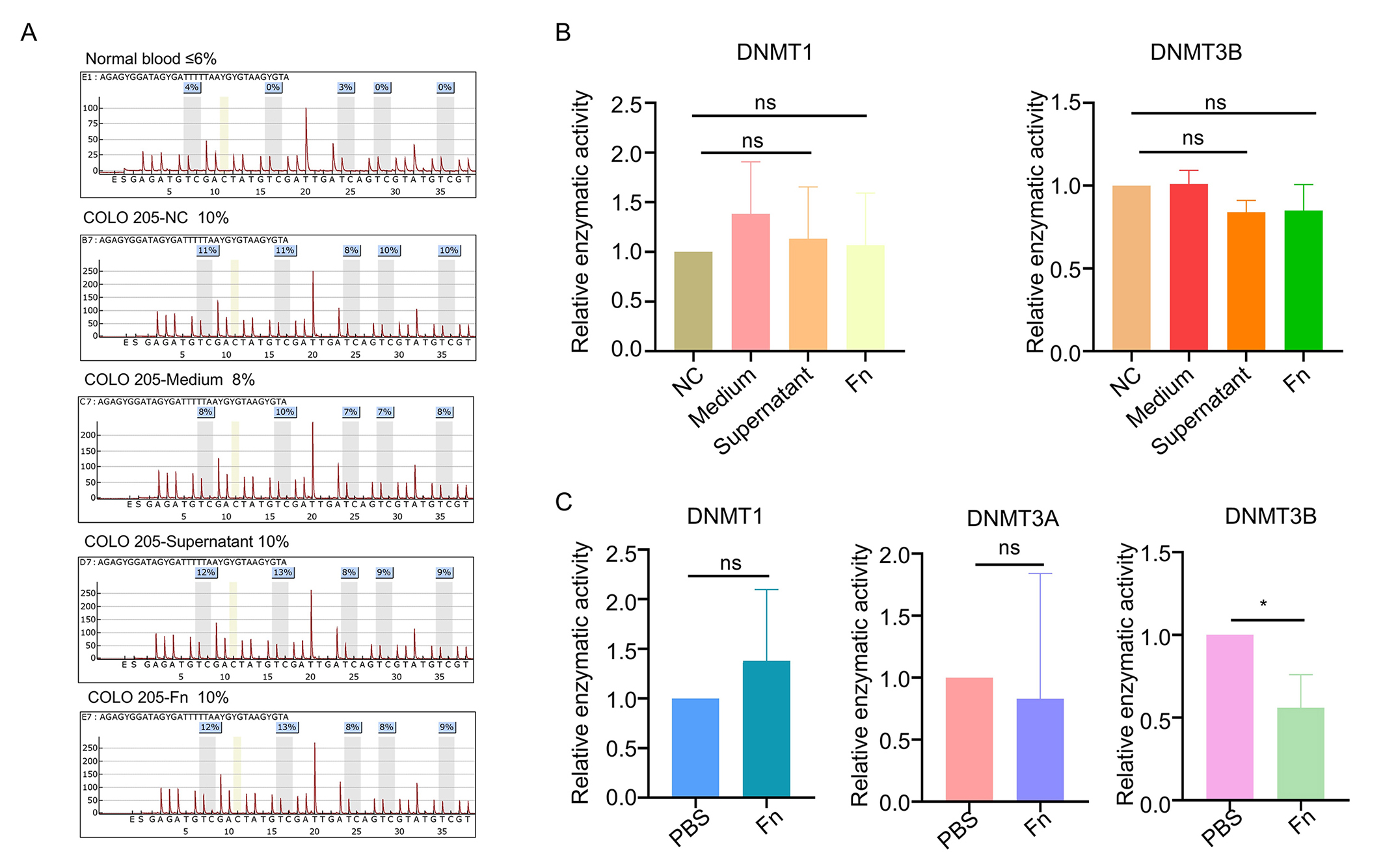

Supplement: Supplementary Figure 3 — Ex-vivo and in-vivo experiments are conducted to investigate the effects of F. nucleatum and its culture supernatant on MLH1 promoter methylation and DNMT activity in COLO 205 cells. (A) The promoter methylation degrees of MLH1 in normal human peripheral blood, untreated COLO 205 cells, blank medium treated COLO 205 cell, supernatant treated COLO 205 cell and F. nucleatum infected COLO 205 cells are ≤ 6%, 10%, 8%, 10% and 10%, respectively. (B) In ex-vivo experiments, there is no difference in the activity of DNMT1 and DNMT3B between the blank medium and F. nucleatum culture supernatant, as well as between F. nucleatum stimulated COLO 205 and the control group. (C) No variations of the enzymatic activities of DNMT1, DNMT3A, and DNMT3B are found in subcutaneous tumors of nude mice from the PBS group and the F. nucleatum infected group. All experiments were independently repeated 3 times. *p<0.05. [file Image3.jpeg]
