# Supplementary material for: Fusobacterium nucleatum downregulated MLH1 expression in colorectal cancer by activating autophagy-lysosome pathway
Source: Front Immunol. 2025 May 19;16:1586146. doi: 10.3389/fimmu.2025.1586146 (PMC12127293; doi:10.3389/fimmu.2025.1586146)
Supplement: Supplementary file 4 [file Table1.docx]

The clinicopathological and molecular characteristics in 567 CRC patients

| Items | Number | Percentage (%) |
| --- | --- | --- |
| Age (years) |  |  |
| <=60 | 131 | 23.1 |
| ＞60 | 436 | 76.9 |
| Gender |  |  |
| Male | 355 | 62.6 |
| Female | 212 | 37.4 |
| Smoking/Drinking history |  |  |
| Present | 161 | 30.8 |
| Absent | 362 | 69.2 |
| Family history |  |  |
| Present | 43 | 7.6 |
| Absent | 520 | 92.4 |
| Differentiation |  |  |
| Poor | 89 | 17.3 |
| Moderate to well | 425 | 82.7 |
| Primary tumor location |  |  |
| Left-sided | 415 | 73.6 |
| Right-sided | 144 | 25.5 |
| Multiple lesions | 5 | 0.9 |
| Tumor size（cm） |  |  |
| <=5 | 393 | 71.3 |
| ＞5 | 158 | 28.7 |
| T staging |  |  |
| T0 | 1 | 0.2 |
| T1 | 34 | 6.2 |
| T2 | 81 | 14.7 |
| T3 | 64 | 11.6 |
| T4 | 371 | 67.3 |
| N staging |  |  |
| N0 | 282 | 51.6 |
| N1 | 164 | 30.0 |

The clinicopathological and molecular characteristics in 567 CRC patients

| Items | Number | Percentage (%) |
| --- | --- | --- |
| N2 | 101 | 18.5 |
| M staging |  |  |
| M0 | 454 | 81.8 |
| M1 | 101 | 18.2 |
| AJCC staging |  |  |
| 0 | 1 | 0.18 |
| I | 84 | 15.3 |
| II | 169 | 30.7 |
| III | 196 | 35.6 |
| IV | 100 | 18.2 |
| Ki-67 |  |  |
| <=65% | 269 | 48.4 |
| ＞65% | 287 | 51.6 |
| MSI status |  |  |
| MSI-H | 30 | 5.4 |
| MSI-L/MSS | 523 | 94.6 |
| *BRAFV600*E mutation |  |  |
| Mutant type | 19 | 3.4 |
| Wild type | 537 | 96.6 |
| *RAS* mutation |  |  |
| Mutant type | 277 | 49.3 |
| Wild type | 285 | 50.7 |
| p53 protein |  |  |
| Positive | 314 | 73.5 |
| Negative | 113 | 26.5 |
| EGFR protein expression |  |  |
| Positive | 159 | 61.9 |
| Negative | 98 | 38.1 |
